# Skin Autofluorescence, a Noninvasive Biomarker of Advanced Glycation End-products, Is Associated With Frailty: The Rotterdam Study

**DOI:** 10.1093/gerona/glac025

**Published:** 2022-01-31

**Authors:** Komal Waqas, Jinluan Chen, Fernando Rivadeneira, André G Uitterlinden, Trudy Voortman, M Carola Zillikens

**Affiliations:** Department of Internal Medicine, Erasmus University Medical Center, Rotterdam, The Netherlands; Department of Internal Medicine, Erasmus University Medical Center, Rotterdam, The Netherlands; Department of Internal Medicine, Erasmus University Medical Center, Rotterdam, The Netherlands; Department of Internal Medicine, Erasmus University Medical Center, Rotterdam, The Netherlands; Department of Epidemiology, Erasmus University Medical Center, Rotterdam, The Netherlands; Department of Epidemiology, Erasmus University Medical Center, Rotterdam, The Netherlands; Division of Human Nutrition and Health, Wageningen University and Research, Wageningen, The Netherlands; Department of Internal Medicine, Erasmus University Medical Center, Rotterdam, The Netherlands

**Keywords:** Frailty index, Physical frailty, Skin advanced glycation end-products

## Abstract

**Background:**

Accumulation of advanced glycation end-products (AGEs) in tissues has been linked to various age-related disease phenotypes. Therefore, we investigated the potential relationship between skin AGE accumulation and frailty.

**Methods:**

A cross-sectional analysis was performed on 2 521 participants from the Rotterdam Study. Skin AGEs were assessed as skin autofluorescence (SAF) using the AGE reader™. We used 2 approaches to define frailty. Fried’s criteria, including weight loss, weakness, slow gait speed, exhaustion, and low physical activity, were used to define physical frailty (presence of ≥3 components) and prefrailty (presence of ≤2 components). Rockwood’s concept, including 38 deficits from physical and psychosocial health domains, was used to calculate the frailty index (score 0–1). Multinomial logistic and multivariate linear regression were used with SAF as exposure and physical frailty (ordinal) and frailty index (continuous) as outcome adjusting for age, sex, diabetes, renal function, socioeconomic status, and smoking status.

**Results:**

The mean SAF was 2.39 ± 0.49 arbitrary units and the median age was 74.2 (14.0) years. Regarding physical frailty, 96 persons (4%) were frail and 1 221 (48%) were prefrail. Skin autofluorescence was associated with both being prefrail (odds ratio [95% confidence interval] = 1.29 [1.07–1.56]) and frail (1.87 [1.20–2.90]) compared with nonfrail. Regarding the frailty index, the median value was 0.14 (0.10–0.19) and higher SAF was also associated with a higher frailty index (coefficient, *B* = 0.017 (0.011–0.023]).

**Conclusions:**

Higher skin AGEs are associated with both physical frailty and frailty index. Longitudinal studies are needed to evaluate the causality and the potential of SAF as a biomarker to screen frailty.

Advanced glycation end-products (AGEs) form a heterogeneous group of compounds that mount up in various tissues as a physiological response to aging. Accumulation of AGEs in tissues such as blood vessels, muscles, bones, and joints (1,2) has been implicated in the pathogenesis of age-related diseases such as cardiovascular disease, sarcopenia, fracture, and osteoarthritis. Exogenous influences such as high-fat, high-protein diet, and smoking (3,4) and endogenous pathologies such as diabetes and chronic kidney disease can expedite the accumulation of AGEs in human tissues (5,6). At the cellular level, AGEs bind to the receptor for AGE (RAGE) resulting in inflammatory/oxidative stress. In the extracellular matrix, AGEs form crosslinks nonenzymatically between protein molecules causing stiffness (7,8). The tissue level of AGEs reflects the body’s longstanding metabolic situation and might play a role in the development of frailty in the older adults (9).

The concept of frailty is evolving as a hallmark of aging in current clinical practice. Frailty is defined as a clinical syndrome of increased vulnerability due to diminished strength, endurance, and reduced physiological function leading to increased dependency (10). Multiple ways to assess frailty are operational at this moment but broadly 2 concepts underlie these approaches; a static stepwise approach including solely physical deficits, that is, physical frailty phenotype originally proposed by Fried (11), and a dynamic continuous approach including cognitive, psychosocial, and physical constellations of deficits, that is, the frailty index originally introduced by Rockwood (12,13). Prevalence of frailty in community-dwelling older adults individuals varies according to the definition used and the subgroups studied (14). Overall, both frailty and AGEs share some common etiology by multisystem involvement during aging and have been reported to predict adverse health outcomes including mortality (15,16).

The relationship between circulating AGEs and physical frailty or its individual components has been analyzed in a few studies (16–18), but AGEs have never been studied in relation to the more comprehensive and dynamic frailty index. A cross-sectional relationship between serum chronic myelogenous leukemia (CML), a noncrosslinking AGE, and physical frailty status was observed, but only among men (19). It is noteworthy that a single-serum AGE might not depict the true burden of tissue AGEs (20) due to both heterogeneity of AGEs and their short half-life in the circulation. A promising, noninvasive technique to estimate tissue AGEs in the skin is the assessment of skin autofluorescence (SAF) using an AGE Reader (21). Skin autofluorescence is considered to be a marker of long-term AGEs burden in the entire body since skin collagen, on which AGEs bind irreversibly, has been shown to have a half-life of nearly 14 years (22). Using SAF as a proxy of tissue AGEs, we presumed that a higher accumulation of tissue AGEs would be involved in these frailty phenotypes.

In the present study, we investigated whether SAF as a surrogate marker of tissue AGEs in middle-aged and older participants of the Rotterdam Study (RS) is associated with physical frailty and the multidimensional frailty index, including both physical and psychosocial deficits, in a cross-sectional manner. We report a significant association between SAF and these 2 frailty phenotypes. Our results suggest a possible etiological role of AGEs in the development of frailty and the potential use of SAF as a biomarker to screen frailty. We call for replication and longitudinal data to confirm our findings.

## Methods

### Study Participants

For this cross-sectional analysis, we included participants from the RS—an ongoing population-based, prospective cohort study as described elsewhere (23). Briefly, participants were included from the Ommoord District in Rotterdam since the outset of the study in 1990. Participants were divided into 3 cohorts based on their year of inclusion, namely RS-I from 1990, RS-II from 2000, and RS-III from 2006. For RS-I and RS-II, all participants aged above 55 years and for RS-III, all participants aged above 45 years were invited for participation. All included participants visited the research center at baseline and every 3–5 years at follow-up examinations. The RS was approved by the institutional review board (Medical Ethics Committee) of Erasmus University Medical Center. All participants provided written informed consent to participate.

In RS, SAF measurement was performed at the sixth follow-up visit of RS-I (2012–2015), fourth follow-up visit of RS-II (2012–2015), and second follow-up visit of RS-III (2011–2013) in 3 027 participants. We included 2 521 participants for final analysis after excluding individuals with no informed consent for follow-up (*n* = 20), outliers of SAF values (*n* = 8), missing data on outcomes namely frailty index and physical activity (PA) (*n* = 408) and on covariates (*n* = 70) including educational level, serum eGFR, smoking, and diabetes status (Supplementary Figure 1).

### Outcomes Frailty

Physical phenotype of frailty and multidimensional frailty index has been the most widely used and validated concepts to analyze frailty in the research (24,25).

### Physical Frailty

Physical frailty was defined using Fried’s criteria (11) as described elsewhere in the RS (26). Physical frailty components were estimated at the same visit as the skin AGE measurement. Briefly, physical frailty was defined as the presence of ≥3 components and prefrailty as the presence of 1 or 2 components from the following criteria:

(1) Weakness was defined using handgrip strength (HGS) that was assessed in the nondominant hand using a hydraulic hand dynamometer (Fabrication Enterprises Inc., White Plains, NY). An HGS of <27 kg for men and <16 kg for women is considered to be a weakness (27).
(2) Weight loss was defined as losing 5% of body weight when compared with a previous follow-up visit about 3–5 years earlier.
(3) Exhaustion was derived from 2 statements from the Center for Epidemiological Studies Depression (CES-D) scale: (a) I felt that everything I did was an effort; (b) I could not get going (28). If these were answered as “frequently” or “mostly”, individuals are considered to be exhausted.
(4) Low PA was defined as ≤14 metabolic equivalent for task (MET) hours per week. The MET is a unit that estimates the amount of energy used by the body during PA, when compared with resting metabolism. Briefly, an adaptive version of the LASA Study Physical Activity Questionnaire was filled in by each participant reporting frequency and duration of different activities in the past 2 weeks (29). The values of MET were assigned to all activities in the questionnaire to quantify activity intensity, using a compendium of activity energy cost (30,31). For example, bicycling to work at a normal pace (<10 mph) has an MET intensity of 4.0 and 1 hour (h) in a week was translated as 4 MET-hours per week.
Slowness was defined using gait speed (GS) that was evaluated using a 5.79-m long walkway (GAITRite Platinum; CIR systems, Sparta, NJ: 4.88-m active area; 120-Hz sampling rate). A subject with a GS of <0.8 m/s was considered to be slow (27).

### Frailty Index

Frailty index was formulated using basic concepts from Rockwood’s approach (13) based on the accumulation of physical, biomedical, and psychosocial health deficits as published elsewhere in the RS (32). Briefly, the frailty index is based on 38 deficits accumulated from 6 major health domains (Supplementary Table 1), namely functional status (*n* = 13), cognition (*n* = 6), diseases (*n* = 6), health conditions (*n* = 6), nutritional status (*n* = 3), and mood (*n* = 4). Every deficit was either dichotomized (yes/no—1/0) or ordinally categorized based on severity (never, sometimes, mostly, always—0, 0.33, 0.66, 1). For every individual, these deficit scores were summed up and divided by a total number of deficits that resulted in a score ranging from 0 (no deficits present, least frail) to 1 (all deficits present) (32).

Frailty index was assessed in RS-III (*n* = 974) participants at the second follow-up visit as was skin AGE measurement. For RS-I (*n* = 614) and RS-II (*n* = 933) participants, frailty index (2010–2012) was estimated during the last follow-up visit before skin AGE measurement (2011–2015).

### Predictor of Interest: SAF

The AGE Reader CU™ ((DiagnOptics Technologies B.V., Groningen, The Netherlands) was introduced in the RS in 2013 to measure SAF as described elsewhere (33). Briefly, a small area of forearm skin, ~4 cm (2), was illuminated with an excitation light source from the AGE Reader with a peak wavelength of 370 nm. The AGE reader utilizes the fluorescent properties of AGEs. It estimates skin AGEs based on the emission and reflection spectrum, which is converted through a software program into numerical values reported in arbitrary units (AU). Thus, an elevated SAF score in AU corresponds to a high tissue AGEs level (21). Automated software in the AGE Reader ensures the incorporation of skin reflectance values between 6% and 10% (corresponds to Fitzpatrick type V skin color) in SAF values and exclusion of participants with skin reflectance under 6% (corresponding to Fitzpatrick type VI or the darkest brown skin color) (34).

### Other Study Parameters

Smoking was obtained through self-reporting by the participants and classified as current, past, or never smokers. Information on the educational level was assessed during the initial interview by trained interviewers according to the UNESCO classification of education (UNESCO, 1976). It contains 4 categories: primary education; lower = intermediate general and lower vocational education; intermediate = higher general and intermediate vocational education; and higher = higher vocational education and university. Height and weight were recorded in standing position at the research center without shoes and BMI was calculated. Type 2 diabetes mellitus (T2DM) was defined by combining the information on antidiabetic medication use, fasting blood glucose levels, and diagnosis in the GP registries (35). Serum creatinine and fasting glucose were measured through an automated enzymatic method. Effective glomerular filtration rate (eGFR) was calculated by the Chronic Kidney Disease Epidemiology Collaboration (CKD-EPI) equation using serum creatinine concentration, age, and sex data (36).

### Statistical Analysis

All statistical analysis was performed using IBM SPSS Statistics 25 (version 25.0). Normality of the residuals of the exposure and predictors of interest was determined using histograms and Shapiro–Wilk test. Data are presented as mean ± standard deviation (*SD*) in case of normal distribution, median (interquartile range [IQR]) in case of non-normal distribution or as count (percentages). Means of continuous variables among groups were compared by using independent samples *t*-test or ANOVA when regression residuals were normally distributed or Mann–Whitney–Wilcoxon test when a non-normal distribution was assumed. A χ ^2^ test was adopted to compare categorical variables.

Binary and multinomial logistic regression was used to investigate the associations between SAF and physical frailty, prefrailty, and its components. Potential confounders were identified based on literature (3,5,6,37). Model 1 included age, sex, and RS-cohorts; Model 2 included in addition effective eGFR, smoking status, diabetes status, educational level, and BMI.

Multiple linear regression analysis was performed to investigate the associations between SAF and frailty index. Potential confounders in this relationship such as diabetes mellitus, BMI, and creatinine were a component of the frailty index (outcome) itself. Therefore, we only included age, sex, RS-cohorts, smoking status, and educational level as covariates. A subgroup analysis was performed in RS-III separately where both SAF and frailty index were measured at the same time point. All regression analysis was performed using the ENTER regression method. Variance inflation factor was checked for every model to check for multicollinearity. We excluded 70 participants with missing data on covariates and no imputation was performed.

#### Sensitivity analysis

Skin autofluorescence levels are influenced by sex, diabetes (38), smoking (39), and decreased renal function (40,41). For physical frailty models, we checked for interaction terms between SAF and diabetes, smoking status, eGFR, and sex in the multivariate models. Subgroup analysis was performed when there was a significant interaction term (*p* ≤ .10). For frailty index, a subgroup analysis was performed after excluding those with T2DM and renal dysfunction (eGFR < 60) as the index itself takes these 2 into account. For PA—an essential component of physical frailty, we used a second definition inculcating a strict threshold of ≤30 MET-hours per week named low PA_30. This was compared with a ≤14 MET-hours per week threshold for calculating physical frailty and its association with SAF.

## Results

### Participant Characteristics

Characteristics of the total population and based on nonfrail, prefrail, and frail for the physical phenotype are given in Table 1. Participants were 74 years old (67–81) and 44% males with a mean SAF value of 2.39 (*SD* = 0.49). For the physical frailty, 1 204 (48%) of the participants had none of the components (nonfrail), 1 221 (48%) had 1 or 2 components (prefrail), and 96 (4%) had ≥3 components (frail). Characteristics for participants above and below the median frailty index value are given in Supplementary Table 2. For the frailty index (0–1), the median value was 0.14 (0.10–0.19). With increasing frailty status, there was a trend toward higher SAF values, older age, female gender, and T2DM, and a trend toward lower eGFR values and educational level (Table 1 and Supplementary Table 2).

**Table 1.** Selected Characteristics for our Total Cohort and Based on Physically Nonfrail, Prefrail, and Frail Individuals

	All	Nonfrail	Prefrail	Physically Frail
Number (%)	2 521	1 204 (48%)	1 221 (48%)	96 (4%)
Frailty index (score 0–1)	0.144 (0.10–0.19)	0.121 (0.08–0.16)	0.158 (0.11–0.21)	0.229 (0.19–0.27)**
Confirmed sarcopenia^a^	86 (3.5%)	NA	70 (6%)	16 (17%)
SAF (AU)	2.39 ± 0.49	2.34 ± 0.46	2.43 ± 0.50	2.58 ± 0.53**
Age (years)	74.2 (14.0)	72.2 (13.2)	75.6 (14.5)	81.3 (9.3)**
Males	1 024 (44%)	567 (47%)	507 (42%)	29 (30%)*
BMI (kg/m^2^)	27.3 ± 4.2	26.8 ± 3.8	27.7 ± 4.5	28.2 ± 4.5**
RS-I/II/III	574/921/850 (25%/39%/36%)	197/467/540 (16%/39%/45%)	366/433/422 (30%/36%/35%)	51/33/12 (53%/34%/13%)**
Energy intake (kcal/day)	2 090.6 (838.5)	2 153.9 (838.3)	2 032.9 (834.5)	1 874.2 (885.5)**
Smokers				*
Never	750 (32%)	415 (35%)	366 (30%)	29 (30%)
Former	1270 (54%)	635 (53%)	655 (54%)	58 (60%)
Current	325 (14%)	154 (13%)	200 (16%)	9 (10%)
eGFR (ml/min/1.73 m2)	77.7 ± 14.5	78.9 ± 13.5	77.4 ± 15.4	72.1 ± 17.6**
Diabetes (T2DM)	306 (13%)	143 (12%)	165 (14%)	22 (23%)*
Education				**
Primary	160 (6%)	60 (5%)	89 (7%)	11 (11.5%)
Lower	986 (39%)	448 (37%)	500 (41%)	38 (40%)
Intermediate	767 (31%)	363 (30%)	372 (30.5%)	32 (33%)
Higher	608 (24%)	333 (28%)	260 (21%)	15 (16%)

*Note:* Data are presented as Mean ± *SD*, median (IQR), and number (%).

SAF = skin autofluorescence; AU = arbitrary units; BMI = body mass index; METh/week = metabolic equivalent task hours per week; kcal/day = kilocalories per day; eGFR = effective glomerular filtration rate; T2DM = type 2 diabetes mellitus; NA = not applicable.

^a^Conformed sarcopenia always includes the presence of weak handgrip strength which is also an overlapping component of physical frailty.

***p <.0001, **p <.001 and *p <.05. *p*-Value for trend based on a statistical comparison of physical frailty categories by ANOVA.

### SAF and Its Association With Physical Frailty Phenotype

**Figure 1.**
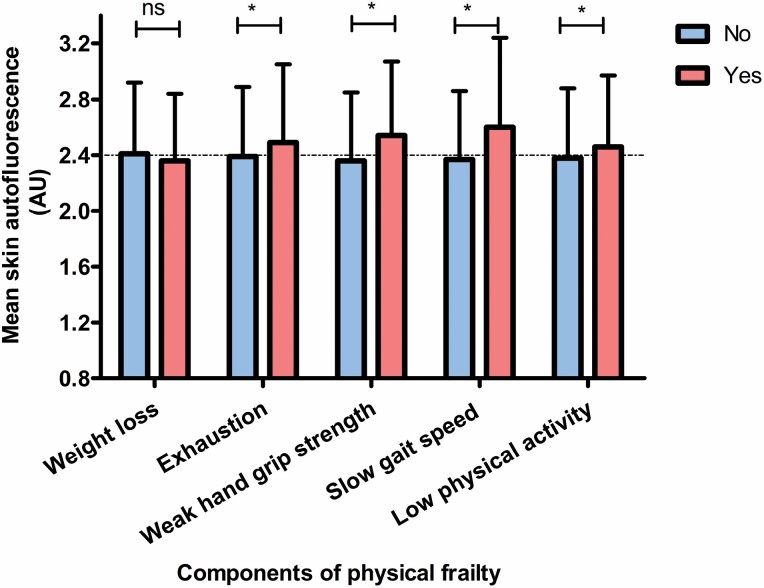
Bar chart showing the skin autofluorescence (SAF) values in relation to the presence or absence of physical frailty components. ns, not significant, *p < .05.

The prevalence of individual components of physical frailty was around 15%–20% except for slow GS which is much less prevalent (3%) (Supplementary Figure 2). The mean SAF values were higher in all individuals with either low PA, exhaustion, slowness, or weakness when compared with normal PA, no exhaustion, no slowness, or no weakness, respectively. The mean SAF values were not different between participants with or without weight loss (Figure 1).

Table 2 shows the results of multivariable logistic regression analysis depicting the association between SAF as a continuous variable and physical frailty and its components as a binary variable. In model 2, higher SAF was significantly associated with higher odds of prevalent exhaustion (odds ratio [OR] = 1.48 per 1 unit higher SAF [95% CI 1.16–1.88], *p* = .002); weak HGS (OR = 1.39 [1.11–1.74], *p* = .004); and slow GS (OR = 1.85 [1.05–3.27], *p* = .03). Skin autofluorescence showed a marginally nonsignificant association with low PA (OR = 1.18 [0.96–1.49], *p* = .13) and no association with ≥5% weight loss in last 5 years (OR = 1.07 [0.82–1.40], *p* = .61).

**Table 2.** Binary Logistic Regression Analysis Between Skin Autofluorescence (SAF) as Exposure and Physical Frailty and Its Components as Outcome

	*N*	Model 1		Model 2	
		Odds Ratio (95% CI)	*p* Value	Odds Ratio (95% CI)	*p* Value
*Nonfrail and prefrail*		Ref.		Ref.	
*Physical frailty*	96/2 521	1.68 (1.11–2.52)	**.01**	1.59 (1.04–2.43)	**.03**
Components of physical frailty					
*Weakness*	583/2 455	1.35 (1.10–1.68)	**.006**	1.39 (1.11–1.74)	**.004**
*Exhaustion*	360/2 513	1.59 (1.26–2.01)	**.0001**	1.48 (1.16–1.88)	**.002**
*Slow gait speed*	52/1 917	1.95 (1.12–3.42)	**.02**	1.85 (1.05–3.27)	**.03**
*Weight loss*	334/2 518	1.17 (0.91–1.52)	.22	1.07 (0.82–1.40)	.61
*Low physical activity*	506/2 521	1.30 (1.05–1.61)	**.01**	*1.18 (0.96–1.49)*	*.13*

*Notes:* Model 1 was adjusted for age, sex, and RS-cohorts. Model 2 was additionally adjusted for eGFR, DM status, smoking status, education level, and BMI. All bold *p*-values are <.05 denoting significant results.

Table 3 shows the results of multinomial logistic regression analysis depicting the association between physical frailty as a ternary dependent variable and SAF as a continuous independent variable. One AU increase in SAF was associated with both being prefrail (OR = 1.29 [1.07–1.56], *p* = .007 or 1 *SD* increase in SAF = 1.14 [1.04–1.25]) and frail (OR = 1.87 [1.20–2.90], *p* = .005 or 1 *SD* increase in SAF = 1.37 [1.10–1.72]) in our model 2 when nonfrail subjects were used as reference.

**Table 3.** Multinomial Logistic Regression Between Skin Autofluorescence (SAF) as Exposure and Prefrailty and Physical Frailty as Outcomes When Compared to Nonfrail Individuals

	Prefrail (*N* = 1 221 or 48%)		Frail (*N* = 96 or4%)		*p* Value of Interaction
	Odds Ratio (95% CI)	*p* Value	Odds Ratio (95% CI)	*p* Value	
	Ref. (nonfrail)		Ref. (nonfrail)		SAF × sex 0.30
Model 1	1.38 (1.15–1.66)	.0004	2.04 (1.34–3.12)	.001	SAF × DM 0.60
Model 2	1.29 (1.07–1.56)	.007	1.87 (1.20–2.90)	.005	SAF × smoking 0.12

*Notes:* Model 1 was adjusted for age, sex, and RS-cohorts. Model 2 was additionally adjusted for eGFR, DM status, smoking status, education level, and BMI. All bold *p*-values are <.05 denoting significant results.

### Skin Autofluorescence and Its Association With Frailty Index

Table 4 shows the results of multivariate associations between SAF and frailty index. Skin autofluorescence was significantly and positively associated with frailty index in the total study population after adjusting for age and sex, RS subcohorts (*B* = 0.019; 95% CI = [0.013, 0.025], *p* = 1.9 × 10^−10^) which attenuated slightly after adjusting for other confounders namely smoking and educational level (*B* = 0.017 [0.011, 0.023], *p* = 1.3 × 10^−10^). In RS-III subcohort, SAF and frailty index were measured cross-sectionally without a time gap between SAF and collection of frailty index components. The association between SAF and frailty index in RS-III showed slightly higher coefficients of association but in the same direction as the total population in our model 2 (*B* = 0.023 [0.013–0.033], *p* = 1.2 × 10^−10^].

**Table 4.** Linear Regression Analysis Between Skin Autofluorescence (SAF) and Frailty Index in the Total Population and for the Subgroup RS-III

	Standardized Coefficient, *B*	Unstandardized Coefficient, *B* (95% CI)	*p* Value
Model 1	0.127	0.019 (0.013–0.025)	1.9 × 10^−10^
Model 2	0.116	0.017(0.011–0.023)	1.3 × 10^−8^
Model 1 (RS-III subgroup)	0.162	0.026 (0.016–0.036)	3.9 × 10^−7^
Model 2 (RS-III subgroup)	0.143	0.023 (0.013–0.033)	1.2 × 10^−5^

*Notes:* Model 1: Frailty index ~ SAF + age + sex + RS-cohorts. Model 2: Model 1 + smoking + educational level. All bold *p*-values are <.05 denoting significant results.

### Subgroup Analysis

For physical frailty, there was no significant interaction term for SAF × sex (*p* =.30), SAF × smoking (*p* = .12), or SAF × CKD (*p* = .37) based on eGFR <60 or ≥60. Stratification by diabetes status showed that the associations of SAF with physical frailty and prefrailty in participants with or without T2DM remained more or less similar with overlapping confidence intervals (Supplementary Table 3).

As the frailty index score includes T2DM and high creatinine as deficits itself, an analysis after excluding those with T2DM and those with eGFR <60 was performed. After exclusion of these participants, there was an attenuation of the association coefficient (*B* = 0.011 [0.004–0.017], *p* = .001) between SAF and frailty index but the relationship remained statistically significant (Supplementary Table 4).

By comparing 2 low PA thresholds, ≤30 or ≤14 MET-hours per week, the prevalence of low PA was 40% or 20%, respectively, which may have increased the prevalence of physical frailty. One unit increase in SAF was associated with a 22% increase in odds of low PA (defined as ≤30 MET-hours per week) after adjustments (1.22 [1.02–1.45], *p* = .03]. This association between SAF and low PA (defined as ≤14 MET-hours per week) was not statistically significant in our model 2 (1.18 [0.96–1.49], *p* = .13) with slight attenuation of mean OR (Supplementary Table 5).

Finally, a subgroup analysis was performed by excluding those with confirmed sarcopenia, which did not change the associations. One AU increase in SAF was associated with both being prefrail (47%) (OR = 1.23 [1.01–1.49], *p* = .04) and frail (3%) (OR = 2.10 [1.28–3.46], *p* = .003) in our model 2 when nonfrail subjects were used as reference (Supplementary Table 6).

## Discussion

Using the physical frailty as defined by Fried, subjects with higher SAF values were more likely to be frail compared with nonfrail participants independent of age and other risk factors. Similar findings were obtained using the frailty index as proposed originally by Rockwood, where the increase in SAF was associated with an increase in frailty index values.

We observed that participants with a 1 unit increase in SAF values were 1.87 times more likely to be frail and 1.29 times to be prefrail compared with nonfrail participants. According to one cross-sectional study in healthy individuals, SAF increased approximately 0.023 AU per year up to 70 years of age (42) which could be translated to approximately 0.3 AU in a decade. In those above 70 years of age and with risk factors for accelerated AGEs accumulation, a much higher rate of increase in SAF (up to 10 times in a year) has been observed (39,43). Keeping this in mind, odds of frailty with one AU increase in SAF seem to be low in our population but in high-risk individuals followed in a temporal fashion, it may become highly relevant. In line with our findings regarding the association of skin AGEs with physical frailty, a higher prevalence of physical frailty with higher serum CML levels was previously observed but only in men and not in women with a mean age of 78 years (comparable age to our cohort) (19). In contrast, in a study including French community-dwelling individuals aged 75 years or older, skin AGEs measured as SAF were neither associated with prevalent (*n* = 71/423 or 16.8%) nor with incident physical frailty (*n* = 32/255 or 12.6%) after 4 years of follow-up. By a closer look, 35% of their participants had chronic kidney disease at baseline when compared with 11% in our cohort. Renal function has been shown to substantially alter the metabolism of both AGEs and frailty status which makes the comparison between the 2 studies difficult. They did observe, however, a positive association of SAF with 2 individual components, namely incident exhaustion and low PA (44). Our study found an association of SAF with the presence of all individual physical frailty components but weight loss.

Frail individuals suffer not only from physical deficits but psychofunctional and communal problems are also quite common. In this respect, frailty index is multidimensional and unique in including cognitive and communal traits in addition to functional parameters. We found that 1 *SD* increase in skin AGEs was associated with a 12% higher frailty index value in our whole cohort and with 7% higher frailty index values after excluding participants with T2DM and eGFR <60 (*n* = 1952). Advanced glycation end-products and frailty may share a common etiology through diverse pathways and multisystem involvement. Firstly, non–enzymatic crosslinking of AGEs between collagen molecules leads to stiffness and alters biomechanical properties of extracellular matrix in, for example, bone, muscles, blood vessels, and joints. Secondly, AGE binding to RAGE leads to activation of pro-inflammatory and oxidative stress pathways that might increase the predisposition to several frailty-related traits (7,8). In line with our hypothesis about potential similar underlying mechanisms between AGEs accumulation and frailty, a recent study in subjects with frailty and cognitive impairment has identified a reduced level of the metabolites related to the antioxidative defense system (45). A panel of frailty biomarkers including three inflammatory markers was recently proposed based on data from gene expression databases. It included interleukin-6 (46) which is one of the key pro-inflammatory cytokines activated through RAGE. In summary, our data corroborate the influence of AGEs in accelerating frailty by negatively influencing a range of parameters.

We recently found that SAF was associated with sarcopenia (47), which increases susceptibility to physical frailty and vice versa. Nonetheless, 82% of the subjects in our cohort categorized as having physical frailty did not have sarcopenia, based on the European working group on Sarcopenia in Older people revised criteria (EWGSOP2) (Table 1). This intraindividual disconcordance between prevalence rates of sarcopenia and frailty has also been reported earlier (48). Although both conditions share some common grounds, frailty is much broader than only musculoskeletal health especially when defined by Rockwood’s concept (13). Therefore, the association of SAF with confirmed sarcopenia and its one component namely weak HGS could not explain the relationship between AGEs and frailty.

We observed that one unit increase in SAF was associated with 18% higher odds of low PA (defined as ≤14 MET-hours per week, prevalence 20%). This relationship attenuated to a nonsignificant level when adjusted for traits such as the presence of T2DM and chronic kidney disease with which AGEs could have a bidirectional relationship (cause or consequence). In line with our observation, Drenth et al. (17) observed that a 1 unit increase in SAF was associated with a 24% higher risk of not complying with the Dutch PA guidelines and 21% lower daily activity, also after adjusting for diabetes, kidney disease, and smoking status. Accumulation of AGEs in skeletal muscle has been implicated in the motor decline and this can lead to low PA. Conversely, low PA augments oxidative and inflammatory stress which increases AGE formation (49). In this situation, a vicious circle initiates between AGE accumulation and low PA (50). Hence, the causal direction of the association between SAF and PA could be bidirectional and difficult to disentangle in this setting.

Current literature about the relationship between AGEs and weight loss is difficult to interpret. Serum AGEs decline during weight loss due to calorie restriction both in subjects with and without diabetes (51,52). On the other hand, skin AGEs did not appear to decrease in those with weight loss after 5 years of follow-up following bariatric surgery (53). The latter was anticipated by authors as skin AGE accumulation is quite stable and determined by collagen turnover which may take up to 14.8 years in the skin (22). Still, unintentional weight loss in the older adults could be a consequence of diverse phenomenona where dietary quality, malnutrition, anorexia of aging, psychological, and cognitive functioning could be listed as top contributors but the list is far-reaching, also including, for example, malignancies (54). Therefore, the absence of an association in our study between SAF and weight loss may be explained by the complex etiology underlying unintentional weight loss in the older adults.

Stratification or subgroup analysis according to diabetes presence showed a similar association of SAF with both physical frailty and frailty index although not always statistically significant in those with T2DM. Importantly, the prevalence of physical frailty was 7% in those with T2DM which is much lower than reported in current literature, that is, up to 30% for T2DM (55). In our population-based cohort, voluntary participation by those with severe phenotypes of T2DM might be low in comparison to studies including participants from out-patient clinics which partially explain the low prevalence of frailty in subjects with T2DM. This inclusion bias might have concealed the association, to some extent, between SAF and frailty in those with T2DM. Finally, whether AGEs accumulation is a risk factor or a consequence of frailty index components such as T2DM and renal function should be a focus of future studies taking temporal associations into account.

Our study has a unique strength to allow us to study both physical and psychosocial components of frailty in relation to skin AGEs. Although residual confounding could not be fully exempted, we were able to adjust for many potential confounders. Limitations are the inclusion of participants only from Dutch background which reduces generalizability of our results. A cross-sectional nature of analysis precludes drawing conclusions about the causal direction of the associations and evaluates its predictive capability. A proportion of subjects had a collection of frailty index components 4–5 years before SAF although a subset had measurements at 1 point of time, showing similar strengths of association (Table 4). Skin autofluorescence measures the fluorescence of skin within a specific wavelength where inclusion of fluorescent non-AGEs compounds and exclusion of nonfluorescent AGEs could not be omitted. Nevertheless, validation studies showed a good correlation between SAF and skin biopsy levels of fluorescent and nonfluorescent AGEs (21). We also could not exclude the possibility of survival bias that could have caused an underestimation of the association between SAF and frailty in our analyzes.

In conclusion, this study demonstrates an association between higher skin AGEs and higher prevalence of physical frailty and overall frailty status indicated by higher values of frailty index. Longitudinal studies are needed to study the causal chain and investigate whether SAF will be useful to identify individuals at risk of developing frailty. On a large-scale, implementation of the noninvasive and rapid SAF measurements can be practically an efficient solution to screen frailty instead of using a panel of complex tests and laborious indices. As frailty development and AGEs accumulation are both considered to be partially preventable and reversible (56,57), studies using interventions including functional diets to reduce oxidative and inflammatory stress or to reduce endogenous AGE burden can yield new prevention strategies.

## Supplementary Material

glac025_suppl_Supplementary_Material
