# Supplementary material for: Skin Autofluorescence, a Noninvasive Biomarker of Advanced Glycation End-products, Is Associated With Frailty: The Rotterdam Study
Source: J Gerontol A Biol Sci Med Sci. 2022 Jan 31;77(10):2032–9. doi: 10.1093/gerona/glac025 (PMC9536452; doi:10.1093/gerona/glac025)

Supplementary data - TABLES

Supplementary Table 1. Overview of the components included in Frailty index (N=38).

**Functional status (*n* = 13)**	**Cognition (*n* = 6)**	**Diseases (*n* = 6)**	**Health conditions (*n* = 6)**	**Nutritional status (*n* = 3)**	**Mood (*n* = 4)**
Dressing, arising,<br><br>Eating, Walking,<br><br>Hygiene, Laundry<br><br>Grip, Riding a bike<br><br>Telephone, Meal<br><br>Gardening, Reach<br><br>Financial	Forgetfulness<br><br>Aphasia<br><br>MMSE<br><br>Stroop<br><br>LDST<br><br>WFT	Cancer<br><br>Lung condition (COPD/Asthma)<br><br>Cardiovascular diseases<br><br>Stroke<br><br>Diabetes Mellitus<br><br>age-related macular degeneration	systolic BP<br><br>falling<br><br>joint<br><br>liver enzymes<br><br>hospital admission<br><br>Creatinine	BMI<br><br>HDL<br><br>Hyperlipidemia	Depressed<br><br>Somatic<br><br>Positive<br><br>Interpersonal

MMSE, Mini-mental state examination; LDST, The Letter Digit Substitution Test; WFT, Word Fluency test; BP, blood pressure; BMI, Body mass index; HDL, high density lipoprotein; COPD, Chronic Obstructive Pulmonary Disease.

Supplementary Table 2. Selected characteristics of our cohort based on an arbitrary cut-off at median value for frailty index.

	**Low Frailty index below median**	**High Frailty index above median**
**N**	1253	1268
**Physical frailty, n (%)**	10 (0.8%)	86 (6.8%)***
**Pre-frailty, n (%)**	497 (39.7%)	724 (57.1%)***
**SAF, AU**	2.30 ± 0.45	2.49 ± 0.52***
**Age (years)**	71.8 (14.6)	77.0 (10.2)
**Males, n (%)**	552 (46%)	472 (41.5%)*
**BMI, kg/m^2**	26.3 ± 3.7	28.3 ± 4.5***
**RS I/II/III, n (%)**	205 / 441 / 607<br>(17% /35% /38%)	409 / 492 / 367<br>(32% /39% /29%)**
**Energy intake, kcal/day**	2123.3 (885.3)	2053.8 (787.7)
**Smokers, n (%)**<br>**Former**<br>**Current**	 *622 (50%)*<br>*197 (16%)*	 *726 (57%)*<br>*166 (13%)*
**eGFR, ml/min per 1.73m^2**	80.7 ± 12.9	76.2 ± 15.4***
**Physical activity, METh/week**	50.0 (67.6)	35.5 (61.5)***
**Diabetes - T2DM, n (%)**	80 (6%)	250 (20%)***
**Education, n (%)**<br>***Primary***<br>***Lower***<br>***Intermediate***<br>***Higher***	 *37 (3%)*<br>*443 (35%)*<br>*395 (32%)*<br>*378 (30%)*	***<br>*123 (10%)*<br>*543 (43%)*<br>*372 (29%)*<br>*230 (18%)*

SAF, skin autofluorescence; AU, arbitrary units; METh/week, metabolic equivalent task hours per week; kcal/day, kilo calories per day; eGFR, effective glomerular filtration rate; T2DM, Type 2 diabetes mellitus.

Data are presented as Mean ± SD, Median (IQR) and number (percentage, %)

*** p<0.0001, * p<0.05. P-value based on a statistical comparison of values between high and low frailty index groups calculated by independent t-test or Mann Whitney.

Supplementary Table 3. Stratified analysis between SAF as exposure and pre-frail and physical frailty as outcomes according to diabetic status.

		Odds ratio (95% CI)	p-value		Odds ratio (95% CI)	p-value
		***T2DM* (n = 330)**			***Non-T2DM* (n=2191)**	
Non-frail	43%	**Ref.**		49%	**Ref.**	
Pre-frail	50%	1.56 (0.94 – 2.58)	0.09	48%	1.25 (1.02 – 1.53)	**0.03**
Frail	7%	2.92 (1.11 – 7.67)	**0.03**	3%	1.58 (0.95 -2.63)	0.08

Model 1 Physical frailty ~ SAF + age + sex + RS-cohorts + eGFR + DM status + smoking status + Education level + BMI

Supplementary Table 4. Analysis between SAF as exposure and frailty index as outcomes after excluding those with type 2 diabetes mellitus and reduced eGFR.

	N	Standardized coefficient, β	Unstandardized coefficient, B (95% CI)	p-value
**Excluding T2DM**	2191	0.088	0.013 (0.007 – 0.019)	**1.9×10^{-5}**
**Excluding eGFR<60**	2235	0.098	0.015 (0.009 – 0.021)	**2×10^{-6}**
**Excluding both**	1952	0.072	0.011 (0.004 – 0.017)	**0.001**

Model 2 Frailty index ~ SAF + age + sex + RS-cohorts + smoking + Educational level

Supplementary Table 5. Analysis between SAF as exposure and low physical activity as outcome (using two thresholds ≤ 30 and ≤ 14 MET hours per week).

	N	**Model 1**		**Model 2**	
		Odds ratio (95% CI)	p-value	Odds ratio (95% CI)	p-value
Low Physical activity_30	990/2521	1.30 (1.09-1.55)	**0.003**	1.22 (1.02-1.45)	**0.03**
Low Physical activity_14	506/2521	1.30 (1.05-1.61)	**0.01**	1.18 (0.96-1.49)	0.13

Low Physical activity_30, less than or equal to 30 metabolic equivalent of task hours per week; Low Physical activity_14, less than or equal to 14 metabolic equivalent of task hours per week.

Model 1 was adjusted for age, sex, and RS-cohorts.

Model 2 was additionally adjusted for eGFR, DM status, smoking status, Education level and BMI

Supplementary Table 6. Multinomial logistic regression between SAF as exposure and pre-frailty and physical frailty as outcomes when compared to non-frail individuals after excluding those with confirmed sarcopenia.

	**Pre-frail (N= 1090 or 47%)**		**Frail (N= 76 or 3%)**	
	Odds ratio (95% CI)	p-value	Odds ratio (95% CI)	p-value
	Ref. (non-frail)		Ref. (non-frail)	
**Model 1**	1.31 (1.08 – 1.58)	**0.005**	2.21 (1.37 – 3.57)	**0.001**
**Model 2**	1.23 (1.01 – 1.49)	**0.04**	2.10 (1.28 – 3.46)	**0.003**

Model 1 was adjusted for age, sex, and RS-cohorts.

Model 2 was additionally adjusted for eGFR, DM status, smoking status, Education level and BMI

Supplementary data – FIGURES

Supplementary Figure 1. Flowchart of participants inclusion from the Rotterdam study.

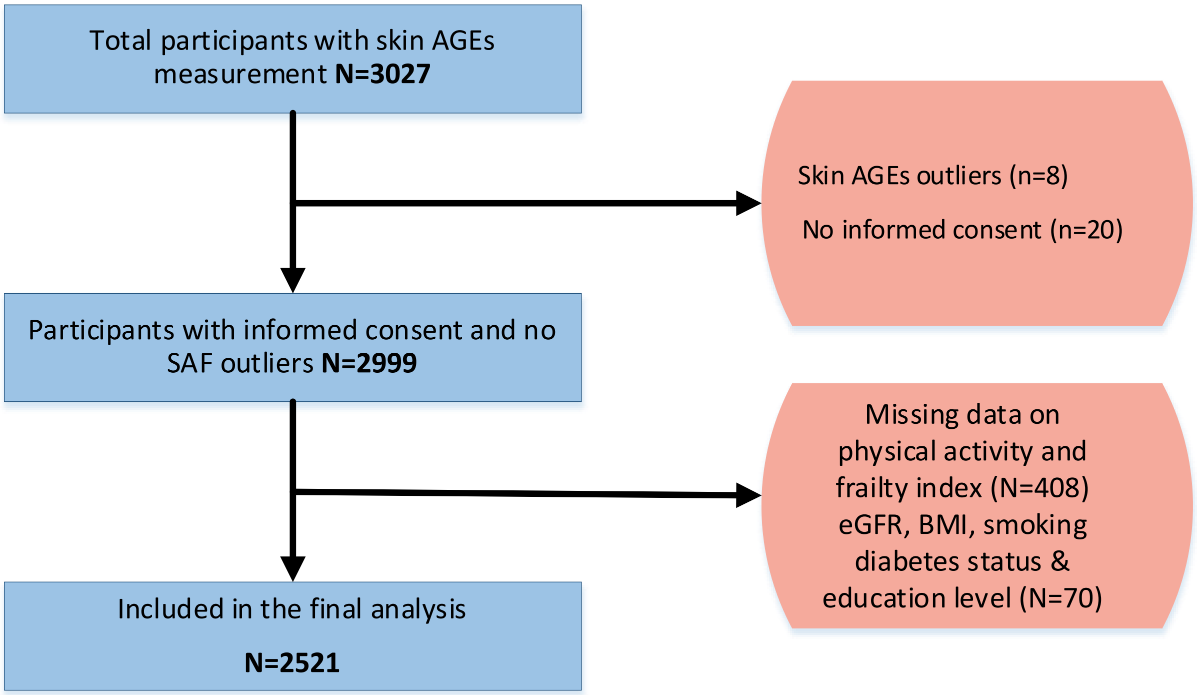

Supplementary Figure 2. Percentage of participants having individual physical frailty components.

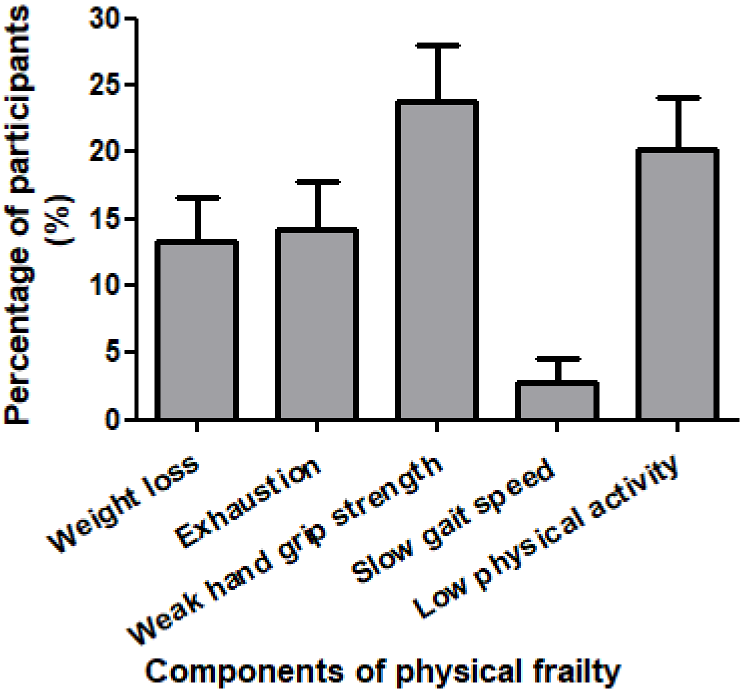

Supplement: glac025_suppl_Supplementary_Material [file glac025_suppl_supplementary_material.pdf]
